# Supplementary material for: Meta-QTL and Candidate Gene Analyses of Agronomic Salt Tolerance and Related Traits in an RIL Population Derived from Solanum pimpinellifolium
Source: Int J Mol Sci. 2024 May 31;25(11):6055. doi: 10.3390/ijms25116055 (PMC11172916; doi:10.3390/ijms25116055)
Supplement: Supplementary file 1 [file ijms-25-06055-s001.zip › ijms-3000776-supplementary.pdf]

Supplementary Materials of article "Meta-QTL and candidate gene analyses of agronomic salt tolerance and related traits in a RIL population derived *from Solanum pimpinellifolium*" by Maria J. Asins* and Emilio A. Carbonell.

Figure S1 Distribution of the 12 MQTL (Table 1) on the linkage map of the 8 chromosomes involved. QTL composition of each MQTL is also specified.

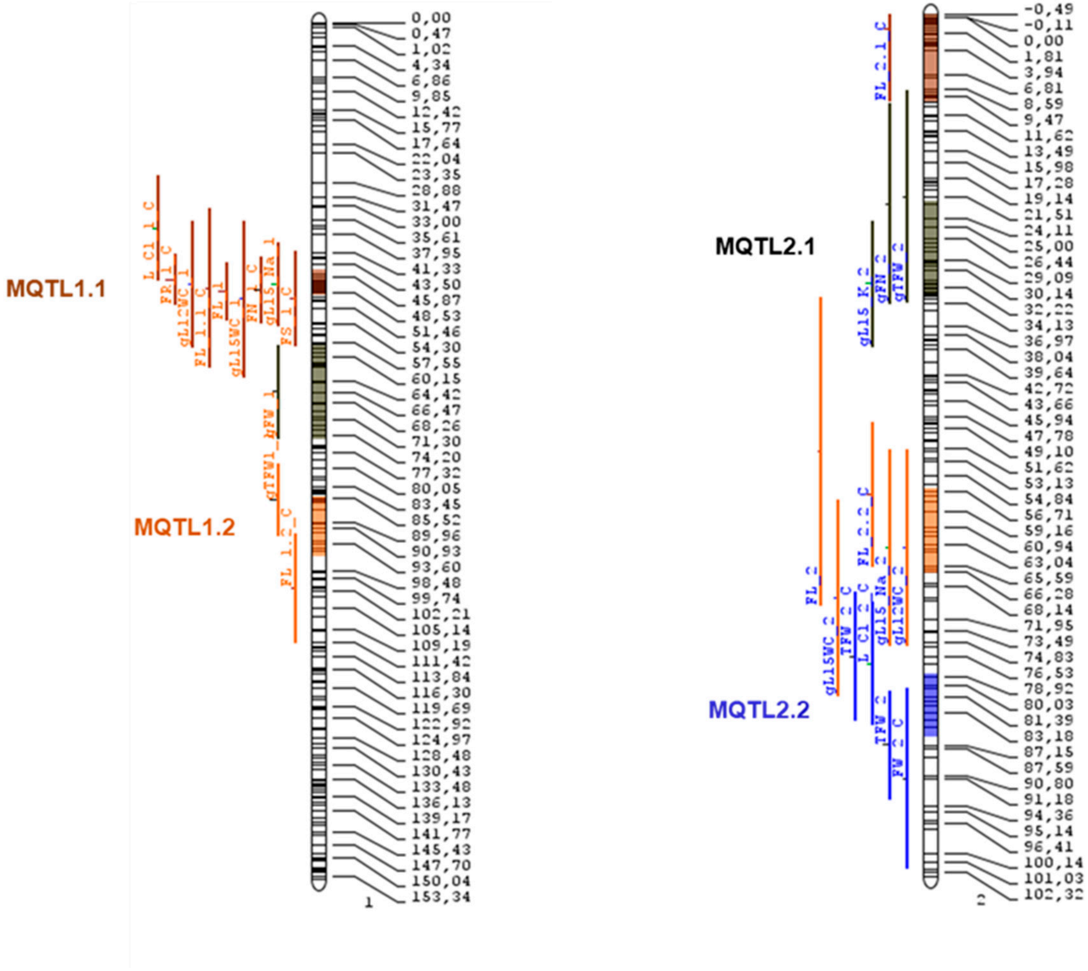

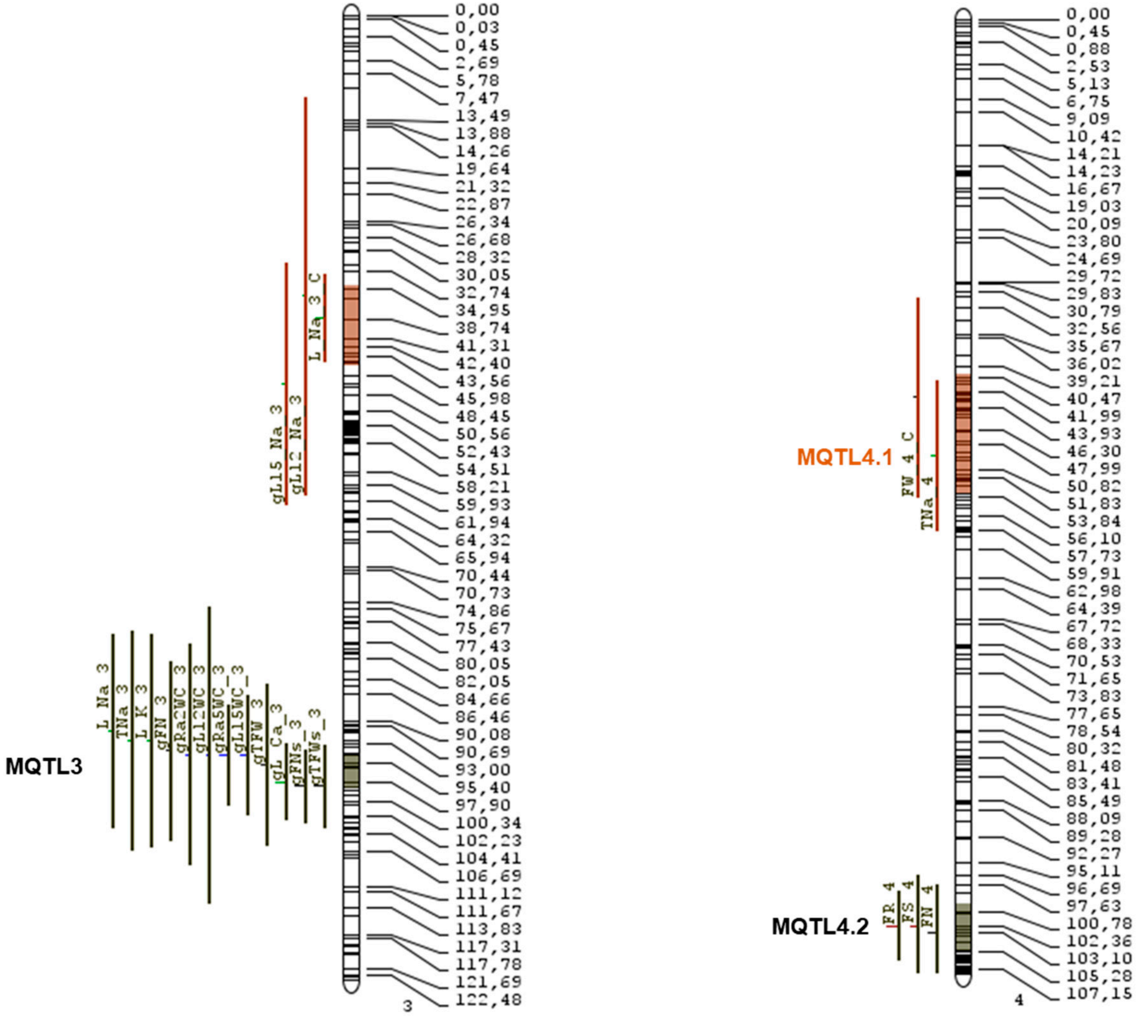
MQTL3
MQTL4.1
MQTL4.2
qL15 Na 3
qL12 Na 3
L Na 3 C
L Na 3
TNa 3
L K 3
qFN 3
qRa2WC 3
qL12WC 3
qRa5WC_3
qL15WC_3
qTFW 3
qL Ca_3
qFNs_3
qTFWs_3
FW 4 C
TNa 4
FR 4
FS 4
FN 4
3
0,00
0,03
0,45
2,69
5,78
7,47
13,49
13,88
14,26
19,64
21,32
22,87
26,34
26,68
28,32
30,05
32,74
34,95
38,74
41,31
42,40
43,56
45,98
48,45
50,56
52,43
54,51
58,21
59,93
61,94
64,32
65,94
70,44
70,73
74,86
75,67
77,43
80,05
82,05
84,66
86,46
90,08
90,69
93,00
95,40
97,90
100,34
102,23
104,41
106,69
111,12
111,67
113,83
117,31
117,78
121,69
122,48
4
0,00
0,45
0,88
2,53
5,13
6,75
9,09
10,42
14,21
14,23
16,67
19,03
20,09
23,80
24,69
29,72
29,83
30,79
32,56
35,67
36,02
39,21
40,47
41,99
43,93
46,30
47,99
50,82
51,83
53,84
56,10
57,73
59,91
62,98
64,39
67,72
68,33
70,53
71,65
73,83
77,65
78,54
80,32
81,48
83,41
85,49
88,09
89,28
92,27
95,11
96,69
97,63
100,78
102,36
103,10
105,28
107,15

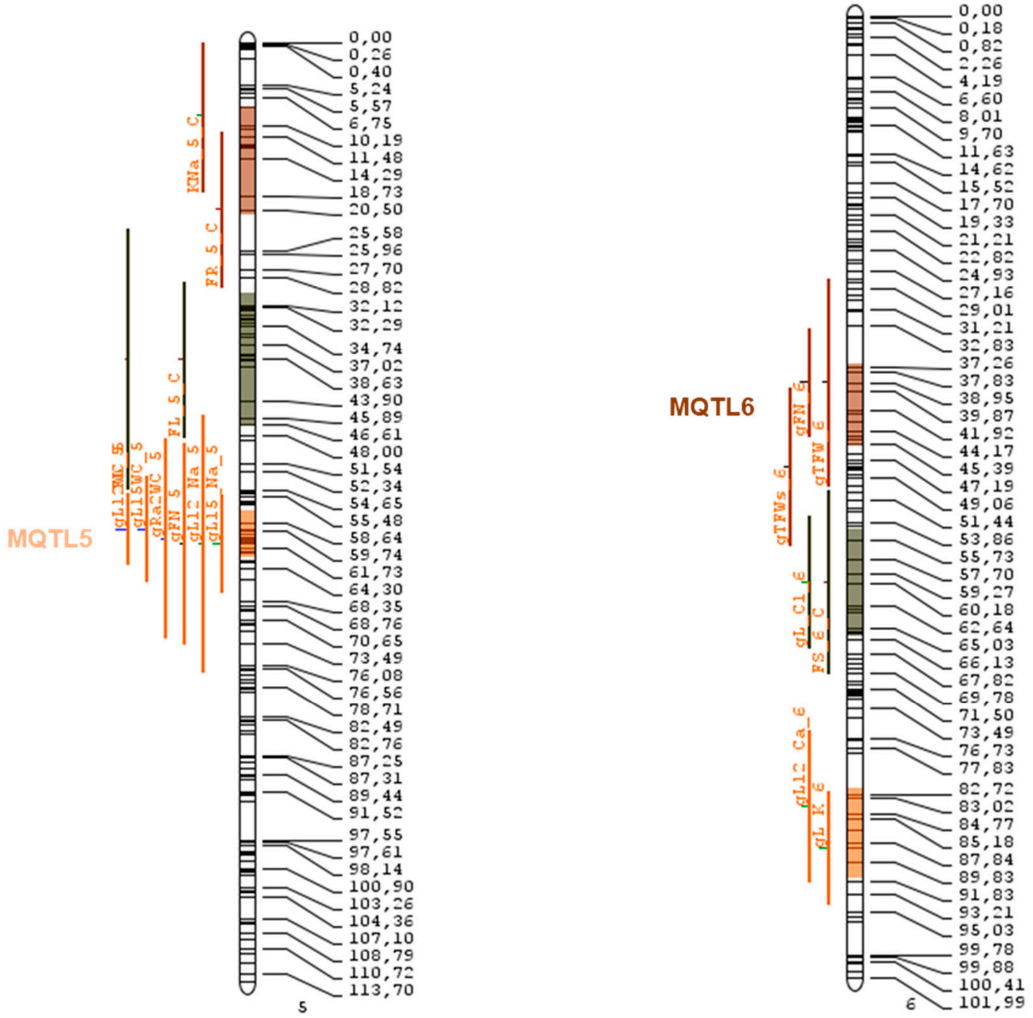
MQTL5
0,00
0,26
0,40
5,24
5,57
6,75
10,19
11,48
14,29
18,73
20,50
25,58
25,96
27,70
28,82
32,12
32,29
34,74
37,02
38,63
43,90
45,89
46,61
48,00
51,54
52,34
54,65
55,48
58,64
59,74
61,73
64,30
68,35
68,76
70,65
73,49
76,08
76,56
78,71
82,49
82,76
87,25
87,31
89,44
91,52
97,55
97,61
98,14
100,90
103,26
104,36
107,10
108,79
110,72
113,70
5
MQTL6
0,00
0,18
0,82
2,26
4,19
6,60
8,01
9,70
11,63
14,62
15,52
17,70
19,33
21,21
22,82
24,93
27,16
29,01
31,21
32,83
37,26
37,83
38,95
39,87
41,92
44,17
45,39
47,19
49,06
51,44
53,86
55,73
57,70
59,27
60,18
62,64
65,03
66,13
67,82
69,78
71,50
73,49
76,73
77,83
82,72
83,02
84,77
85,18
87,84
89,83
91,83
93,21
95,03
99,78
99,88
100,41
101,99
6

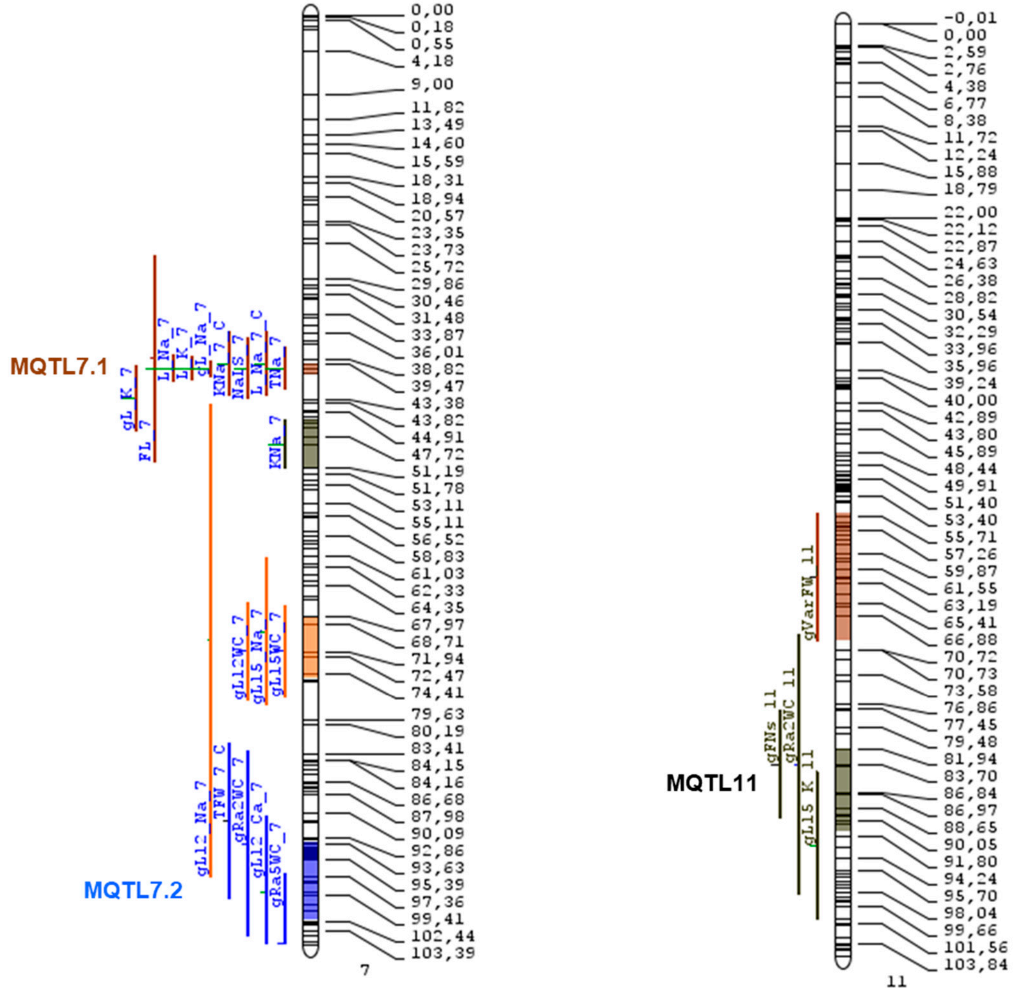
MQTL7.1
MQTL7.2
gL_K_7
FL_7
L_Na_7
L_K_7
gL_Na_7
KNa_7_C
NaUS_7
L_Na_7_C
TNa_7
KNa_7
gL12WC_7
gL15_Na_7
gL15WC_7
gL12_Na_7
TFW_7_C
gRa2WC_7
gL12_Ca_7
gRa5WC_7
0,00
0,18
0,55
4,18
9,00
11,82
13,49
14,60
15,59
18,31
18,94
20,57
23,35
23,73
25,72
29,86
30,46
31,48
33,87
36,01
38,82
39,47
43,38
43,82
44,91
47,72
51,19
51,78
53,11
55,11
56,52
58,83
61,03
62,33
64,35
67,97
68,71
71,94
72,47
74,41
79,63
80,19
83,41
84,15
84,16
86,68
87,98
90,09
92,86
93,63
95,39
97,36
99,41
102,44
103,39
7
MQTL11
gVarFW_11
gFNs_11
gRa2WC_11
gL15_K_11
-0,01
0,00
2,59
2,76
4,38
6,77
8,38
11,72
12,24
15,88
18,79
22,00
22,12
22,87
24,63
26,38
28,82
30,54
32,29
33,96
35,96
39,24
40,00
42,89
43,80
45,89
48,44
49,91
51,40
53,40
55,71
57,26
59,87
61,55
63,19
65,41
66,88
70,72
70,73
73,58
76,86
77,45
79,48
81,94
83,70
86,84
86,97
88,65
90,05
91,80
94,24
95,70
98,04
99,66
101,56
103,84
11

Table S1. List of QTL and their characteristics: Trait type (Ion, WC: water content, FY: fruit yield, Rep: reproductive), location and year of experiment, position (Pos.) in cM, chromosome (Chr.), LOD (logarithm of the odds) score, r^2 (the amount of variation accounted for), number of the nearest solcap_snp_sl marker (SNP), confidence interval presented as the left (CI_lo) and right (CI_hi) genetic positions in cM, and the increasing trait allele (I.Ale, p from *S. pimpinellifollium*, l from *S. lycopersicum*). The reference reporting the salt tolerance experiment is also included.

QTL	Trait Type	Location	Year	Chr	LOD	r^2	Pos	SNP	CI_Lo	Lo_SNP	CI_hi	hi_SNP	I.Ale	Ref.
**gRa2WC_7**	Water	Murcia	2007	7	3,00	0,06	93,63	70865	83,12	6313	104,15	70564	p	Asins et al. 2010
**gRa2WC_5**	Water	Murcia	2007	5	2,57	0,05	60,65	37122	48,38	50701	72,92	22609	e	Asins et al. 2010
**gRa2WC_9**	Water	Murcia	2007	9	2,47	0,05	45,68	45078	32,69	39735	58,67	43039	e	Asins et al. 2010
**gRa2WC_3**	Water	Murcia	2007	3	2,27	0,05	94,40	CL015369-0414	80,31	58651	108,50	61634	p	Asins et al. 2010
**gRa2WC_11**	Water	Murcia	2007	11	2,18	0,05	83,71	56295	68,98	CL009240-0441	98,43	2733	e	Asins et al. 2010
**gLl2WC_5**	Water	Murcia	2007	5	7,59	0,15	59,49	51111	55,01	5050	63,97	CL017077-0623	e	Asins et al. 2010
**gLl2WC_7**	Water	Murcia	2007	7	6,21	0,12	71,94	53489	66,32	6370	77,55	6338	p	Asins et al. 2010
**gLl2WC_1**	Water	Murcia	2007	1	3,21	0,06	47,13	70345	35,70	59944	58,55	38126	p	Asins et al. 2010
**gLl2WC_2**	Water	Murcia	2007	2	3,11	0,06	63,52	SGN-U316197	51,68	49664	75,35	CL015559-0344	e	Asins et al. 2010
**gLl2WC_9**	Water	Murcia	2007	9	2,28	0,04	115,11	63713	98,55	69633	131,68	63588	p	Asins et al. 2010
**gLl2WC_10**	Water	Murcia	2007	10	2,12	0,04	83,18	8780	65,27	CritIso_Promoter_SNP1	101,08	8780	p	Asins et al. 2010
**gLl2WC_3**	Water	Murcia	2007	3	2,01	0,04	94,40	CL015369-0414	75,47	35697	113,34	34015	p	Asins et al. 2010
**gRa5WC_3**	Water	Murcia	2007	3	3,95	0,10	94,40	CL015369-0414	87,97	9382	100,84	61875	p	Asins et al. 2010
**gRa5WC_7**	Water	Murcia	2007	7	3,27	0,08	104,90	37031	97,01	70865	112,78	37031	p	Asins et al. 2010
**gLl5WC_7**	Water	Murcia	2007	7	6,33	0,13	71,94	53489	66,72	6370	77,15	6338	p	Asins et al. 2010
**gLl5WC_5**	Water	Murcia	2007	5	5,23	0,10	59,49	51111	53,00	CL015186-0095	65,99	30	e	Asins et al. 2010
**gLl5WC_3**	Water	Murcia	2007	3	4,47	0,09	94,40	CL015369-0414	86,70	9382	102,11	61847	p	Asins et al. 2010
**gLl5WC_9**	Water	Murcia	2007	9	3,32	0,06	115,11	63713	104,59	69697	125,63	63588	e	Asins et al. 2010
**gLl5WC_2**	Water	Murcia	2007	2	2,99	0,06	69,51	42411	57,68	SGN-U586520	81,34	36273	e	Asins et al. 2010
**gLl5WC_1**	Water	Murcia	2007	1	2,51	0,05	49,84	50504	35,74	59944	63,93	38346	p	Asins et al. 2010
**gLl2_Na_5**	Ion	Murcia	2007	5	2,51	0,04	61,28	51142	45,58	48839	76,98	22621	e	Asins et al. 2010
**gLl2_Na_3**	Ion	Murcia	2007	3	4,22	0,03	35,95	41379	10,46	63301	61,43	35313	p	Asins et al. 2010

gLl2_Na_7	Ion	Murcia	2007	7	2,60	0,02	70,71	53489	43,89	55136	97,54	70865	p	Asins et al. 2010
gLl5_Na_10	Ion	Murcia	2007	10	5,93	0,12	81,86	60745	76,10	CL017176-0241	87,62	8780	p	Asins et al. 2010
gLl5_Na_5	Ion	Murcia	2007	5	5,73	0,11	61,28	51142	55,31	50844	67,25	16140	e	Asins et al. 2010
gLl5_Na_1	Ion	Murcia	2007	1	4,62	0,09	47,13	70345	39,60	59913	54,66	25914	p	Asins et al. 2010
gLl5_Na_7	Ion	Murcia	2007	7	4,25	0,08	69,71	53534	61,33	14183	78,10	53387	p	Asins et al. 2010
gLl5_Na_2	Ion	Murcia	2007	2	3,03	0,06	63,52	SGN-U316197	51,68	49664	75,35	CL015559-0344	e	Asins et al. 2010
gLl5_Na_3	Ion	Murcia	2007	3	2,40	0,04	47,10	26385	31,69	54887	62,51	35313	p	Asins et al. 2010
gLl5_Na_9	Ion	Murcia	2007	9	2,29	0,04	71,92	CL016636-0429	56,14	65898	87,69	58299	e	Asins et al. 2010
gLl5_K_2	Ion	Murcia	2007	2	2,65	0,09	31,93	25401	24,31	CL009018-1241	39,55	29720	e	Asins et al. 2010
gLl5_K_11	Ion	Murcia	2007	11	2,45	0,08	92,94	56127	84,66	56286	101,22	28473	e	Asins et al. 2010
gLl2_Ca_6	Ion	Murcia	2007	6	2,86	0,08	84,02	57217	75,94	57377	92,10	54473	e	Asins et al. 2010
gLl2_Ca_7	Ion	Murcia	2007	7	2,69	0,08	99,08	CL017605-0415	90,47	31930	107,68	37031	e	Asins et al. 2010
gLl2_Ca_8	Ion	Murcia	2007	8	2,50	0,07	102,05	65541	92,71	65287	111,38	41942	p	Asins et al. 2010
**gFN_2**	Yield	Murcia	2007	2	2,36	0,06	22,39	10557	10,34	8405	34,43	13638	e	Estañ et al. 2008
**gFN_3**	Yield	Murcia	2007	3	2,48	0,06	93,90	62270	82,48	58518	105,33	34106	p	Estañ et al. 2008
**gFN_5**	Yield	Murcia	2007	5	2,34	0,05	61,28	51142	49,01	50701	73,55	22609	e	Estañ et al. 2008
**gFN_6**	Yield	Murcia	2007	6	4,77	0,12	38,95	55909	33,19	SL10768_412	44,72	56045	p	Estañ et al. 2008
**gFW_1**	Yield	Murcia	2007	1	2,58	0,08	66,47	38406	57,97	38096	74,96	27452	p	Estañ et al. 2008
**gTFW_3**	Yield	Murcia	2007	3	2,78	0,06	95,71	62120	85,36	62544	106,06	34106	p	Estañ et al. 2008
**gTFW_6**	Yield	Murcia	2007	6	2,65	0,06	38,95	55909	27,91	68851	50,00	41811	p	Estañ et al. 2008
**gTFW_2**	Yield	Murcia	2007	2	2,28	0,05	21,51	33633	8,77	67520	34,25	13638	e	Estañ et al. 2008
FN_1_C	Yield	Valencia	2001	1	3,18	0,09	48,20	13762	42,10	59890	54,31	37999	p	Villalta et al. 2007
FN_12_C	Yield	Valencia	2001	12	1,94	0,06	77,63	55627	67,56	42740	87,70	32706	p	Villalta et al. 2007
FW_2_C	Yield	Valencia	2001	2	2,02	0,05	91,18	66953	80,35	67361	102,01	48033	e	Villalta et al. 2007
FW_4_C	Yield	Valencia	2001	4	1,93	0,05	42,75	45517	31,49	21390	54,00	24660	e	Villalta et al. 2007
TFW_2_C	Yield	Valencia	2001	2	2,59	0,07	76,53	29914	68,77	42441	84,28	67117	e	Villalta et al. 2007
TFW_7_C	Yield	Valencia	2001	7	2,27	0,07	91,07	SGN-U564051	82,24	6313	99,90	70741	e	Villalta et al. 2007
FN_4	Yield	Valencia	2001	4	3,91	0,11	103,10	3841	97,63	47487	108,57	4148	p	Villalta et al. 2007
TFW_2	Yield	Valencia	2001	2	2,71	0,09	87,15	67052	80,63	67361	93,67	66878	e	Villalta et al. 2007

FL_2.1_C	Reproductive	Valencia	2001	2	3,56	0,07	1,43	Le006654_46	-0,49	26013	10,13	8405	p	Villalta et al. 2007
FL_2.2_C	Reproductive	Valencia	2001	2	3,54	0,07	57,15	SGN-U586520	48,45	49576	65,84	42537	p	Villalta et al. 2007
FL_5_C	Reproductive	Valencia	2001	5	3,23	0,06	38,80	23711	29,23	48962	48,37	50701	p	Villalta et al. 2007
FL_1.2_C	Reproductive	Valencia	2001	1	3,14	0,06	101,74	43857	91,85	27964	111,64	43902	p	Villalta et al. 2007
FL_1.1_C	Reproductive	Valencia	2001	1	2,25	0,04	47,90	50576	33,55	59975	62,25	38346	p	Villalta et al. 2007
FR_1_C	Reproductive	Valencia	2001	1	4,42	0,12	46,29	59784	41,66	59897	50,91	50427	p	Villalta et al. 2007
FR_5_C	Reproductive	Valencia	2001	5	2,16	0,06	20,50	49084	10,94	52811	30,07	48962	p	Villalta et al. 2007
FS_1_C	Reproductive	Valencia	2001	1	2,38	0,07	49,84	50504	41,27	59897	58,40	38126	p	Villalta et al. 2007
FS_6_C	Reproductive	Valencia	2001	6	2,10	0,06	60,18	42119	50,45	41840	69,91	39395	p	Villalta et al. 2007
L_Cl_2_C	Ion	Valencia	2001	2	2,90	0,08	77,39	49752	70,04	42383	84,75	67090	e	Villalta et al. 2007
L_Cl_1_C	Ion	Valencia	2001	1	2,25	0,06	37,06	20438	27,49	60089	46,62	50427	p	Villalta et al. 2007
FL_1	Reproductive	Valencia	2001	1	6,88	0,11	48,53	50539	43,27	33677	53,80	SL20127_1427	p	Villalta et al. 2007
FL_8	Reproductive	Valencia	2001	8	3,86	0,06	77,18	34761	67,29	64564	87,08	15436	p	Villalta et al. 2007
FL_7	Reproductive	Valencia	2001	7	3,32	0,05	38,82	67869	27,10	2819_5_183_b	50,53	67766	e	Villalta et al. 2007
FL_5	Reproductive	Valencia	2001	5	2,45	0,04	38,80	23711	22,86	49084	54,74	50775	p	Villalta et al. 2007
FL_2	Reproductive	Valencia	2001	2	2,11	0,03	51,92	49669	33,41	29543	70,44	42383	p	Villalta et al. 2007
FR_4	Reproductive	Valencia	2001	4	5,45	0,15	102,36	47656	98,48	47540	106,23	4051	p	Villalta et al. 2007
FS_4	Reproductive	Valencia	2001	4	3,46	0,10	102,36	47656	96,56	47439	108,15	4148	p	Villalta et al. 2007
L_Na_7_C	Ion	Malaga	2003	7	6,81	0,16	39,47	57002	35,83	67911	43,10	38698	p	Villalta et al. 2008
L_Na_3_C	Ion	Malaga	2003	3	4,61	0,10	38,74	41389	33,17	41324	44,31	26323	e	Villalta et al. 2008
L_Na_10_C	Ion	Malaga	2003	10	2,59	0,05	37,10	62952	25,84	52085	48,35	33007	e	Villalta et al. 2008
KNa_7_C	Ion	Malaga	2003	7	5,43	0,16	39,47	56997	35,83	67911	43,10	38698	e	Villalta et al. 2008
KNa_5_C	Ion	Malaga	2003	5	2,33	0,06	8,95	52748	0,00	69165	18,36	49127	e	Villalta et al. 2008
L_Na_7	Ion	Malaga	2003	7	14,16	0,35	39,96	57007	38,34	67869	41,59	45589	p	Villalta et al. 2008
L_Na_3	Ion	Malaga	2003	3	2,46	0,05	91,37	SL10206_98	78,90	19654	103,85	9140	p	Villalta et al. 2008
L_K_7	Ion	Malaga	2003	7	18,22	0,43	39,96	57007	38,62	67869	41,31	45589	e	Villalta et al. 2008
L_K_8	Ion	Malaga	2003	8	2,44	0,04	0,00	CL016245-0267	0,00	CL016245-0267	13,35	56631	p	Villalta et al. 2008
L_K_3	Ion	Malaga	2003	3	2,37	0,04	92,57	62300	78,90	19654	106,23	61721	e	Villalta et al. 2008
KNa_7	Ion	Malaga	2003	7	8,13	0,21	48,53	38951	45,77	CL015605-0403	51,29	67766	e	Villalta et al. 2008

KNa_8	Ion	Malaga	2003	8	2,51	0,06	19,12	19807	9,23	56575	29,02	56922	p	Villalta et al. 2008
TNa_7	Ion	Malaga	2003	7	11,67	0,24	39,96	57007	37,57	67896	42,35	38698	p	Villalta et al. 2008
TNa_4	Ion	Malaga	2003	4	3,75	0,07	49,34	53136	40,77	41623	57,90	3106	p	Villalta et al. 2008
TNa_3	Ion	Malaga	2003	3	2,33	0,04	92,56	62362	78,56	19654	106,56	61721	p	Villalta et al. 2008
NaLS_7	Ion	Malaga	2003	7	4,15	0,17	39,96	57007	36,51	67905	43,42	38698	e	Villalta et al. 2008

Table S2. Details of the MQTL gene-enrichment analyses where some pathways were significant (FDR: False Discovery Rate). The majority increasing allele (P from *S. pimpinellifollium,* L from *S. lycopersicum*) is indicated between parenthesis after the MQTL name. QTLs at each MQTL are also indicated. When the increasing allele of one or more of them is different from the majority MQTL increasing allele, it is indicated by the corresponding letter.

MQTL & QTLs	**Pathway**	Enrichment FDR	# genes	Pathway genes	Fold Enrichment
**MQTL 1.1 (P)**	RNA phosphodiester bond hydrolysis, endonucleolytic	5.8E-20	17	43	35.4
	RNA phosphodiester bond hydrolysis	2.9E-18	17	54	28.2
**FL_1.1_C**	Production of siRNA involved in RNA interference	1.5E-16	13	28	41.6
**FL_1**	RNA interference	3.4E-16	13	30	38.9
**FN_1_C**	Nucleic acid phosphodiester bond hydrolysis	4.6E-16	17	74	20.6
**FR_1_C**	DsRNA processing	1.2E-14	13	39	29.9
**FS_1_C**	Production of small RNA involved in gene silencing by RNA	1.2E-14	13	39	29.9
**gLI2WC_1**	Post-transcriptional gene silencing by RNA	6.4E-14	13	44	26.5
**gLI5_Na_1**	Posttranscriptional gene silencing	1.1E-13	13	46	25.3
**gLI5WC_1**	Gene silencing by RNA	3E-12	14	73	17.2
**L_Cl_1_C**	Gene silencing	7.1E-12	14	78	16.1
	RNA processing	3.4E-11	30	546	4.9
	Posttranscriptional reg. of gene expression	3.2E-09	14	122	10.3
	Neg. reg. of gene expression	0.00000014	14	163	7.7
	Neg. reg. of macromolecule metabolic proc.	0.0000033	17	316	4.8
	Respiratory electron transport chain	0.000027	8	70	10.2
	Electron transport chain	0.000027	12	184	5.8
	Endosperm development	0.00054	3	6	44.8
	ATP synthesis coupled electron transport	0.00065	6	55	9.8
	Oxidative phosphorylation	0.0012	6	62	8.7
**MQTL 1.2 (P)**	Spliceosomal tri-snRNP complex assembly	5.40E-04	2	6	395.7
	Spliceosomal snRNP assembly	4.50E-03	2	23	103.2

FL_1.2_C	RNA modification	2.50E-02	3	266	13.4
gTFWl_1	Ribonucleoprotein complex assembly	4.00E-02	2	133	17.9
	Ribonucleoprotein complex subunit organization	4.00E-02	2	134	17.7
	RNA splicing, via transesterification reactions	4.00E-02	2	138	17.2
	RNA splicing, via transesterification reactions with bulged adenosine as nucleop	4.00E-02	2	138	17.2
	MRNA splicing, via spliceosome	4.00E-02	2	129	18.4
	RNA splicing	4.60E-02	2	157	15.1
**MQTL 3 (P)**	RNA modification	0.0015	4	266	27.2
gFN_3	Reg. of transport	0.0260	2	56	64.7
gFNs_3	Reg. of localization	0.0260	2	69	52.5
gLl2WC_3	Reg. of metal ion transport	0.0500	1	3	604
gLl5WC_3					
gRa2WC_3					
gRa5WC_3					
gTFW_3					
gTFWs_3					
L_K_3 (L)					
gL_Ca_3					
L_Na_3					
TNa_3					
**MQTL 4 (P)**	Pos. reg. of transcription, DNA-templated	0.0120	3	246	17.5
	Pos. reg. of RNA biosynthetic proc.	0.0120	3	246	17.5
FN_4	Pos. reg. of nucleic acid-templated transcription	0.0120	3	246	17.5
FR_4	Pos. reg. of nucleobase-containing compound metabolic proc.	0.0120	3	283	15.2
FS_4	Anaerobic respiration	0.0200	1	3	478.2
	Histone H4-K16 acetylation	0.0200	1	4	358.6
	Histone H4-K8 acetylation	0.0220	1	5	286.9
	Histone H4-K5 acetylation	0.0250	1	6	239.1
	Histone H4 acetylation	0.0290	1	8	179.3

	Response to auxin	0.0380	2	193	14.9
**MQTL 5 (L)**	Protein autophosphorylation	0.000028	5	110	33.3
**gFN_5**	Chromatin organization	0.00061	4	114	25.7
**gLl2_Na_5**	Chromosome organization	0.0082	4	248	11.8
**gLl2WC_5**					
**gLl5_Na_5**					
**gLl5WC_5**					
**gRa2WC_5**					
**MQTL 2.2 (L)**	Amine transport	0.000000002	4	8	419.9
	Reg. of organic acid transport	0.000000002	4	8	419.9
**FW_2_C**	Amino acid export across plasma membrane	0.000000002	4	8	419.9
**L_Cl_2_C**	Reg. of amine transport	0.000000002	4	8	419.9
**TFW_2**	Reg. of amino acid export	0.000000002	4	8	419.9
**TFW_2_C**	Export across plasma membrane	0.000000017	4	13	258.4
	Nitrate import	0.000000360	3	6	419.9
	Reg. of root development	0.000000570	3	7	359.9
	Reg. of shoot system morphogenesis	0.000000770	3	8	314.9
	Reg. of leaf morphogenesis	0.000000770	3	8	314.9
	Reg. of plant organ morphogenesis	0.000002100	3	11	229
	Nitrate transport	0.000002600	3	12	209.9
	Amino acid transmembrane transport	0.000002700	4	50	67.2
**MQTL 7.2 (P)**	Cellular response to nitrogen starvation	0.031000	2	10	101.3
	Response to salicylic acid	0.031000	2	25	40.5
**gRa2WC_7**	Fruit development	0.031000	3	99	15.3
**gRa5WC_7**	Response to osmotic stress	0.031000	3	106	14.3
**TFW_7_C (L)**	Reproductive structure development	0.031000	4	228	8.9
**gLl2_Ca_7 (L)**	Reproductive system development	0.031000	4	228	8.9
	Developmental proc. involved in reproduction	0.031000	4	251	8.1

Reproductive proc.	0.031000	5	385	6.6
Reproduction	0.031000	5	389	6.5
Cell communication	0.031000	8	843	4.8
Transcription, DNA-templated	0.031000	10	1680	3
Nucleic acid-templated transcription	0.031000	10	1682	3
RNA biosynthetic proc.	0.031000	10	1686	3
Response to starvation	0.032000	2	32	31.6
Response to external stimulus	0.038000	4	280	7.2
Post-embryonic development	0.038000	4	285	7.1
Cellular response to nutrient levels	0.042000	2	41	24.7
Reg. of primary metabolic proc.	0.042000	10	1819	2.8

Table S3. Summary list of candidate genes (mRNA) within each MQTL and their expression (in RPKM) in root (R), Flower (Fl) and leaf (L) of *S. lycopersicum* cv. Heinz (Hei) and *S. pimpinellifolium* LA1589 (Pim). QTLs at each MQTL are also indicated. The majority increasing allele (P from *S. pimpinellifollium*, L from *S. lycopersicum*) is indicated between parenthesis after the MQTL name. When the increasing allele of one or more of them is different from the majority MQTL increasing allele, it is indicated by the corresponding letter. Mutated candidate genes at the *S. pimpinelifolium* (L5) or the *S. lycopersicum* (E9) allele is indicated in the description column.

MQTL	Descrption	mRNA	Hei_R	Pim_R	Hei_Fl	Pim_Fl	Hei_L	Pim_L
**1.1 (P)**	Porin/voltage-dependent anion-selective channel protein	Solyc01g010760	88.3	69.8	93.8	93.1		
	Dehydration-responsive family protein	Solyc01g010870	25.4	32.0	14.8	9.9		
	E3 ubiquitin-protein ligase MARCH3	Solyc01g010880	11.1	14.0	77.6	134.5		
	Major latex-like protein	Solyc01g011470	0.0	0.9	0.0	0.0		
	S-adenosyl-L-methionine salicylic acid carboxyl methyltransf.	Solyc01g014320	0.0	0.0	1.0	0.3		
	S-adenosyl-L-methionine carboxyl methyltransferase family protein	Solyc01g014330	0.1	0.0	0.3	0.1		
	Ethylene insensitive 3 class transcription factor	Solyc01g014480	16.7	17.6	11.1	10.5		
	ARID/BRIGHT DNA-binding domain-containing protein	Solyc01g017030	0.0	0.0	0.0	0.1		
	ARID/BRIGHT DNA-binding domain-containing protein	Solyc01g017790	0.0	0.0	0.0	0.1		
	Unknown Protein (IPR001463 Sodium:alanine symporter)	Solyc01g034180	0.0	0.0	0.0	0.0		
	Unknown Protein (IPR001463 Sodium:alanine symporter)	Solyc01g034220	0.0	0.0	0.0	0.0		
	**Aquaporin SIP12 (*SlSIP2.1*)**	Solyc01g056720	40.2	52.6	30.2	30.4		
FL_1.1_C	Ethylene-responsive transcription factor 13	Solyc01g057080	0.0	4.1	0.0	0.0		
FL_1	AE family transporter anion exchange	Solyc01g057770	14.4	9.2	5.0	10.4		
FN_1_C	Gibberellin 2-oxidase	Solyc01g058030	0.6	1.4	0.0	0.0		
FR_1_C	Gibberellin 2-oxidase	Solyc01g058040	0.0	0.9	0.0	0.0		
FS_1_C	Gibberellin 3-beta-hydroxylase	Solyc01g058250	0.2	2.7	0.9	0.8		
gLl2WC_1	Response regulator 8	Solyc01g065540	19.4	17.4	10.5	8.1		
gLl5_Na_1	Protein DEHYDRATION-INDUCED 19 homolog 4	Solyc01g065550	7.1	10.8	22.3	33.5		
gLl5WC_1	**Flowering promoting factor-like 1 (Fragment)**	Solyc01g066970	73.3	353.9	13.6	13.5		
L_Cl_1_C	**Flowering promoting factor-like 1 (Fragment)**	Solyc01g066980	36.9	47.3	0.9	0.5		

	Ethylene responsive transcription factor 12	Solyc01g067540	3.4	3.5	0.0	0.0
	**Ethylene responsive transcription factor 2b (Sl-ERF.E.4, ERF6)**	Solyc01g065980	224.9	206.4	98.4	51.8
	NaCl-inducible Ca2+-binding protein	Solyc01g058720	4.9	67.0	71.0	121.2
	HB-HD-ZIP	Solyc01g010600	0.2	0.1	4.2	13.0
	bHLH	Solyc01g057300	0.0	0.0	0.1	0.2
	GRAS	Solyc01g059950	0.8	1.0	0.5	0.0
	33x Ulp1 protease family					
	44x Dicer-like protein					
	4x1-AMINOCYCLOPROPANE-1-CARBOXYLATE OXIDASE-like protein					
**1.2 (P)**	**LBD18 LOB domain protein 30**	Solyc01g091420	2.8	3.5		
	Response regulator 10	Solyc01g091810	0.0	0.0		
	Ubiquitin-related modifier 1 homolog	Solyc01g091890	22.8	35.3		
	Gibberellin 20-oxidase 4	Solyc01g093980	0.1	0.1		
**FL_1.2_C**	MYB transcription factor	Solyc01g094360	0.0	0.0		
**gTFWl_1**	Remorin 2	Solyc01g094370	72.4	48.9		
	**Gibberellin 20-oxidase-like protein**	Solyc01g094590	4.1	8.4		
	**Aquaporin (*SlPIP1.2*)**	Solyc01g094690	134.4	28.2		
	Ubiquitin-conjugating enzyme	Solyc01g094810	87.6	99.0		
	ARID/BRIGHT DNA-binding domain-containing protein	Solyc01g094820	8.1	12.4		
	**Ferric reductase oxidase**	Solyc01g094890	41.7	46.9		
	Ferric reductase	Solyc01g094900	2.9	0.4		
	**Ferric reductase oxidase Fro1**	Solyc01g094910	6.3	0.7		
	E3 ubiquitin-protein ligase RNF5	Solyc01g095090	1.3	0.4		
	**Ubiquitin-conjugating enzyme E2 8**	Solyc01g095490	35.9	76.2		
	Ethylene-responsive transcription factor 4	Solyc01g095500	1.0	0.6		
	GH3 family protein (early auxine-responsive gene)	Solyc01g095580	29.1	26.4		
	**Abscisic acid receptor PYL8 (L5)**	Solyc01g095700	76.9	145.5		

	O-methyltransferase family 3 (L5)	Solyc01g095920	0.0	0.0				
	E3 ubiquitin-protein ligase sina	Solyc01g096020	29.5	15.0				
	**Auxin response factor 18 (Sl-ARF18)**	Solyc01g096070	100.6	75.3				
**3 (P)**	Peroxin Pex14 (L5)	Solyc03g114330	10.4	10.3				
	1-deoxy-D-xylulose-5-phosphate reductoisomerase DXR (L5)	Solyc03g114340	31.5	70.1				
**gFN_3**	Ethylene-responsive transcription factor 3	Solyc03g114440	4.1	3.3				
**gFNs_3**	Extracellular calcium sensing receptor	Solyc03g114450	2.9	12.5				
**gLl2WC_3**	Heat shock protein DnaJ domain protein (E9)	Solyc03g114470	20.6	41.8				
**gLl5WC_3**	MADS box transcription factor (SlMBP7e/LeFUL2)	Solyc03g114830	0.2	0.2				
**gRa2WC_3**	MADS box transcription factor 11 (LOC543884)	Solyc03g114840	0.0	0.1				
**gRa5WC_3**	**Cytochrome P450 SlKLUH (fw3.2)**	Solyc03g114940	10.8	1.2				
**gTFW_3**	Expansin (L5)	Solyc03g115300	0.0	0.0				
**gTFWs_3**	Expansin (L5)	Solyc03g115310	0.0	0.0				
**L_K_3 (L)**	Expansin (L5)	Solyc03g115320	0.0	0.0				
**gL_Ca_3**	Eukaryotic translation initiation factor 5A (E9)	Solyc03g115650	198.0	201.1				
**L_Na_3**	**Expansin 10 (L5)**	Solyc03g115890	4.9	6.2				
**TNa_3**	MADS-box transcription factor 1 (L5)	Solyc03g115910	0.0	1.4				
	E3 ubiquitin-protein ligase MARCH3	Solyc03g116030	33.5	39.0				
	3x Chaperone							
**4.1**	**AN1-type Zn finger prot.**	Solyc04g015570	5.7	6.5	13.9	37.2	3.4	2.0
	**Kinesin-4**	Solyc04g016080	1.4	1.0	0.1	0.1	0.1	1.3
**FW_4_C (L)**	Sodium/hydrogen exchanger Na+ H+ antiporter	Solyc04g018090	3.3	2.0	1.1	2.4	1.3	3.0
**TNa_4 (P)**	Sodium/hydrogen exchanger Na+ H+ antiporter 7/8	Solyc04g018100	1.5	2.0	1.3	2.4	1.3	2.1
	Sodium/calcium exchanger family protein (L5)	Solyc04g039960	0.3	0.0	0.0	0.6	0.0	0.7
	**NHX2**	Solyc04g056600	24.1	29.0	14.3	14.5	9.4	5.1
	Auxin Efflux Carrier	Solyc04g056620	9.2	2.1	0.0	0.0	0.0	0.0
	**CLV2**	Solyc04g056640	2.3	3.2	1.0	1.5	1.2	0.8
	Ethylene receptor	Solyc04g025660	nd	0.0	nd	0.0	nd	0.0

**4.2 (P)**	Ornithine carbamoyltransferase	Solyc04g080610	29.3	45.7	17.3	36.6		
	Cytokinin oxidase/dehydrogenase	Solyc04g080820	2.3	61.3	10.4	57.8		
**FN_4**	Ethylene-responsive transcription factor 13	Solyc04g080910	0.0	0.0	0.0	0.0		
**FR_4**	Purple acid phosphatase (L5)	Solyc04g080920	0.0	0.2	0.5	15.0		
**FS_4**	MADS box transcription factor MADS-MIKC	Solyc04g081000	0.5	0.1	543.2	457.9		
	**Cell growth defect factor 2**	Solyc04g081160	18.3	21.5	12.0	20.8		
	**Auxin response factor 5 Sl-ARF5)**	Solyc04g081240	15.2	48.6	7.5	29.3		
	Auxin-induced SAUR-like protein ((early auxine-responsive gene)	Solyc04g081250	3.9	0.8	1.6	1.3		
	Auxin-induced SAUR-like protein (early auxine-responsive gene)	Solyc04g081270	0.2	0.5	2.0	1.8		
	HB-BELL	Solyc04g080790	29.8	161.9	13.9	50.7		
	DBB	Solyc04g081020	13.3	183.7	0.3	2.8		
**5 (L)**	Pto-like serine/threonine kinase (fen)	Solyc05g013290	8.7	7.3				
	Pto-like serine/threonine kinase (pto)	Solyc05g013320	14.3	11.7				
	Primary amine oxidase	Solyc05g013440	217.7	152.3				
**gFN_5**	Phosphate transporter 2-1	Solyc05g013510	0.2	12.5				
**gLl2_Na_5**	Unknown protein (Fragment) (Similar a holotricin-3 like)	Solyc05g013630	548.6	713.9				
**gLl2WC_5**	Auxin-independent growth protein (Fragment)	Solyc05g013730	26.4	27.3				
**gLl5_Na_5**	Abc transporter family protein	Solyc05g013900	0.1	0.0				
**gLl5WC_5**	Multidrug resistance protein ABC transporter family	Solyc05g014380	2.8	70.6				
**gRa2WC_5**	ABC transporter C family member 3	Solyc05g014390	3.7	12.3				
	Multidrug resistance protein ABC transporter family	Solyc05g014500	15.6	2.9				
	**Cell division protein kinase 10**	Solyc05g014760	54.0	150.1				
	**Alpha-glucosidase I (L5)**	Solyc05g015250	12.6	3.2				
	**Myb family transcription factor**	Solyc05g013420	0.1	0.0				
	3x Ulp1 protease family C							
**7.1 (P)**	**HKT1;2 Potassium transporter**	Solyc07g014680	44.5	1.1	0.0	3.3	3.1	1.3
**FL_7 (L)**	**HKT1;1 Potassium transporter**	Solyc07g014690	0.7	159.3	0.8	1.2	0.1	1.2

L_Na_7_C	Bile acid sodium symporter	Solyc07g014740	3.5	12.1	12.2	19.0
KNa_7_C (L)	Decarboxylase (L5)	Solyc07g016080	0.0	0.0	15.2	9.7
L_Na_7	**Auxin response factor 7A (Sl-ARF7A)**	Solyc07g016180	25.9	42.2	26.6	79.9
L_K_7 (L)	Receptor like kinase%2C RLK (LePRK2 PRK2)	Solyc07g017230	0.1	0.3	101.7	3243.9
TNa_7	Pseudouridine synthase (L5)	Solyc07g017250	5.9	0.8	3.0	0.6
NaLS_7 (L)	Lariat debranching enzyme (L5)	Solyc07g017350	3.2	0.0	0.8	0.4
gL_Na_7	Receptor-like protein kinase At3g21340 (L5)	Solyc07g017550	0.0	0.2	0.0	0.1
gL_K_7 (L)						
**11 (L)**	**Sl-ARF4 Auxin response factor 4**	Solyc11g069190	27.6	35.2		
	**Sl-ARF10A Auxin response factor 10A**	Solyc11g069500	13.3	79.7		
**gFNs_11**	Transcription factor MADS-box	Solyc11g069770	2.1	2.8		
gRa2WC_11	**Cytokinin riboside monophosphate phosphoribohydrolase LOG**	Solyc11g069570	4.4	22.6		
gLl5_K_11	**Aquaporin (SlPIP2.6)**	Solyc11g069430	3219.0	548.1		
	ATP-binding cassette protein	Solyc11g069090	442.6	228.2		
	ABC transporter G family member 3	Solyc11g069710	11.5	12.0		
	**Nitrate transporter**	Solyc11g069740	208.7	0.1		
	Nitrate transporter	Solyc11g069750	2.1	0.6		
	**High affinity nitrate transporter protein**	Solyc11g069760	93.4	2.8		
**2.1 (L)**	**Solute carrier family 12 (Potassium/chloride transporters) member 6**	Solyc02g070290	11.8	11.4		
	**Potassium channel**	Solyc02g070530	0.2	0.1		
	**Outward rectifying potassium channel**	Solyc02g071140	11.2	10.0		
**gTFW_2**	Response regulator 5	Solyc02g071220	10.8	17.6		
**gFN_2**	1-aminocyclopropane-1-carboxylate oxidase 1 (L5)	Solyc02g071410	12.3	7.4		
gLl5_K_2	Brassinosteroid signaling positive regulator-related protein	Solyc02g071990	19.2	32.4		
	Aquaporin Z-water channel protein (*SlNIP2.2*)	Solyc02g071910	0.0	0.0		
	**Transmembrane water channel Aquaporin Z (*SlNIP1.2*)**	Solyc02g071920	13.9	11.0		
	Receptor like kinase%2C RLK (L5)	Solyc02g072310	6.7	3.3		

	General vesicular transport factor p115 (E9)	Solyc02g075610	62.3	113.6				
	**Abscisic acid receptor PYL6**	Solyc02g076770	0.2	0.0				
	Cathepsin B-like cysteine proteinase Rcr3 (L5)	Solyc02g076980	65.9	8.8				
	Ethylene responsive transcription factor 1b (Sl-ERF.B.2)	Solyc02g077360	0.5	1.4				
	**Ethylene-responsive transcription factor 2 (Sl-ERF.C.5 PTI5)**	Solyc02g077370	4.3	70.3				
	NADH ubiquinone oxidoreductase complex I (L5)	Solyc02g072160	1.2	17.8				
	Lipid A export ATP-binding/permease protein msbA (L5)	Solyc02g071340	0.0	0.0				
	2x NAD-dependent epimerase/dehydratase (L5)							
	9x FAD-binding domain-containing protein (L5)							
	3x Subtilisin-like serine protease (L5)							
**2.2 (L)**	ima matriochka (Zinc finger-homeodomain protein 1)	Solyc02g087970	2.8	1.0	258.3	587.7		
	EXPA5 Expansin	Solyc02g088100	13.8	10.2	63.3	56.2		
	Organic anion transporter	Solyc02g088900	13.4	9.9	3.1	8.4	7.2	8.3
**FW_2_C**	**TM29 MADS-box transcription factor**	Solyc02g089200	0.5	0.6	373.5	289.8		
**L_Cl_2_C**	SlMBP20 MADS box transcription factor	Solyc02g089210	6.9	1.6	0.8	4.7		
**TFW_2**	Pollen Ole e 1 allergen and extensin	Solyc02g089250	305.1	9.2	56.8	341.5		
**TFW_2_C**	GAST1 Gibberellin regulated protein	Solyc02g089350	4.0	1.1	80.6	190.0		
	Oxygen-evolving enhancer protein 1 of photosystem II	Solyc02g090030	2.2	22.7	349.6	179.0		
	Cyclin-dependent kinase inhibitor 7	Solyc02g090680	3.4	9.9	11.9	13.0	12.1	14.5
	**ORFX Cell number regulator 1 (fruit weight 2.2)**	Solyc02g090730	2.9	2.2	0.2	0.2	0.0	0.1
	Multidrug resistance protein mdtK	Solyc02g090740	2.8	2.8	4.3	1.1	3.5	1.0
	CYP85A1 CYP85 D Cytochrome P450	Solyc02g089160	2.0	3.6	12.6	30.8		
**6 (P)**	**SlPIN6 Auxin Efflux Facilitator**	Solyc06g059730	1.4	0.2				
	**1-aminocyclopropane-1-carboxylate oxidase**	Solyc06g060070	17.1	3.7				
	SlNAM1 NAC domain protein IPR003441	Solyc06g060230	13.1	46.3				
**gFN_6**	**Abscisic acid receptor PYR1**	Solyc06g061180	56.2	25.1				
**gTFW_6**	Early flowering 3	Solyc06g062480	3.0	6.3				
**gTFWs_6**	**Gibberellin 2-beta-dioxygenase**	Solyc06g060800	4.4	26.9				
	**Nitrate transporter NPF6.3**	Solyc06g060620	8.7	22.8				

	Nodulin MtN3 family protein	Solyc06g060590	8.4	19.6
	**Trehalose 6-phosphate phosphatase**	Solyc06g060600	17.7	7.9
	**TIP1 Aquaporin (*SlTIP2.3*)**	Solyc06g060760	2123.7	576.7
**7.2 (P)**	**NOTABILIS/LeNCEDI (9-cis-epoxycarotenoid dioxygenase**	Solyc07g056570	3.7	87.4
	**Ethylene receptor ETR2**	Solyc07g056580	10.6	35.7
	Glucan synthase like 3 (L5)	Solyc07g061920	13.1	59.7
**gRa2WC_7**	Integral membrane single C2 domain protein (L5)	Solyc07g061950	33.4	39.7
**gRa5WC_7**	Mitogen-activated protein kinase 18 (L5)	Solyc07g062080	23.9	49.7
**TFW_7_C (L)**	RAB3 GTPase activating protein subunit 2 (L5)	Solyc07g062450	7.4	21.2
**gLl2_Ca_7 (L)**	Cytochrome P450 (L5)	Solyc07g062500	19.8	189.8
	Serine/threonine phosphatase family protein DIG3(L5)	Solyc07g062970	10.8	36.2
	Stig1 (Fragment)	Solyc07g063090	13.4	0.2
	Root cap protein 2	Solyc07g063490	21.4	1.4
	CHP-rich zinc finger protein-like (L5)	Solyc07g063680	93.7	41.0
	**Indole-3-acetic acid-amido synthetase GH3.8**	Solyc07g063850	7.5	121.2
	GRAS family transcription factor GRAS2	Solyc07g063940	11.8	68.8
	Ubiquitin (L5)	Solyc07g064130	3137.0	5396.2
	Translation initiation factor SUI1 (L5	Solyc07g064150	729.2	1028.7
	**Geranylgeranyl pyrophosphate synthase (E9)**	Solyc07g064660	0.7	0.2
	Beta-1 3-galactosyltransferase 6 (E9)	Solyc07g064670	45.6	8.1
	Small ubiquitin-related modifier (L5)	Solyc07g064880	46.6	178.7
	S-adenosyl-L-methionine salicylic acid carboxyl methyltransf.	Solyc07g064990	2.1	13.2
	Polygalacturonase inhibitor protein (L5)	Solyc07g065090	25.0	15.1

Table S4. Details of reported QTL studies used for present MQTL analysis. Type of marker used for linkage map, NaCl treatments expressed as the electrical conductivity of the final nutrient solution in dS/m. Location and year of the experiment and evaluated traits. Traits as named in original references. Fruit yield traits in black, reproductive traits in brown, leaf ion traits in green and water content traits in blue. When the traits were evaluated in the RIL population as rootstocks, a "g" is added before the trait code.

**Ref.**	**Villalta et al. 2007**	**Villalta et al. 2008**	**Estañ et al. 2008**	**Asins et al. 2010**	**Asins et al 2015**
**Marker**	SSR	SSR	SSR	SSR	SNP
**NaCl Treat. (dS/m)**	0.3, 15	0.3, 9.5	13.7	13.7	8.9
**Location**	Valencia	Malaga	Murcia	Murcia	Valencia
**Year**	2001	2003	2007	2007	2013
**Grafted**	N	N	Y	Y	Y
**Traits**	**FN**<br>**FW**<br>**TFW**<br>**FL**<br>**FR**<br>**FS**<br>**L_Cl**	**L_Na**<br>**L_K/Na**<br>**L_K**<br>**TNa**<br>**NaLS**	**gFN**<br>**gFW**<br>**gTFW**	**gR2WC**<br>**gLi2WC**<br>**gRa5WC**<br>**gLl5WC**<br>**gLl2_Na**<br>**gLl5_Na**<br>**gLl5_K**<br>gLl2_Ca	**gFNs**<br>**gTFWs**<br>**gTFWl**<br>**gL_Na**<br>**gL_K**<br>**gL_Cl**<br>**gL_Ca**

Table S5. BioMercator QTL file.

mapName=E9xL5RI0

**gFNs_11**	**Yield**	null	Valencia	2013	11	11	3,14	0,108	83,7	77,61427718	89,78572282
**gFNs_3**	**Yield**	null	Valencia	2013	3	3	4	0,139	98,4	93,67152472	103,1284753
**gTFWs_3**	**Yield**	null	Valencia	2013	3	3	3,53	0,124	98,4	93,09953174	103,7004683
**gTFWs_6**	**Yield**	null	Valencia	2013	6	6	2,16	0,078	48	39,57361456	56,42638544
**gTFWl_1**	**Yield**	null	Valencia	2013	1	1	2,86	0,101	85,9	79,39249441	92,40750559
**gVarFW_11**	**Yield**	null	Valencia	2013	11	11	2,26	0,091	62,6	55,37738391	69,82261609
**gL_Na_7**	**Ion**	null	Valencia	2013	7	7	30,58	0,682	40	39,0362785	40,9637215
**gL_K_6**	**Ion**	null	Valencia	2013	6	6	3,07	0,109	88,4	82,3701095	94,4298905
**gL_K_7**	**Ion**	null	Valencia	2013	7	7	5,64	0,175	43,4	39,64423963	47,15576037
**gL_Cl_6**	**Ion**	null	Valencia	2013	6	6	2,49	0,093	60,2	53,13270898	67,26729102
**gL_Ca_3**	**Ion**	null	Valencia	2013	3	3	4,18	0,134	97,904	92,99908907	102,8089109
**FN_1_C**	**Yield**	null	Valencia	2001	1	1	3,18	0,094	48,202	42,09621636	54,30778364
**FN_12_C**	**Yield**	null	Valencia	2001	12	12	1,94	0,057	77,626	67,55681295	87,69518705
**FW_2_C**	**Yield**	null	Valencia	2001	2	2	2,02	0,053	91,181	80,3518743	102,0101257
**FW_4_C**	**Yield**	null	Valencia	2001	4	4	1,93	0,051	42,748	31,49420271	54,00179729
**TFW_2_C**	**Yield**	null	Valencia	2001	2	2	2,59	0,074	76,525	68,76900457	84,28099543
**TFW_7_C**	**Yield**	null	Valencia	2001	7	7	2,27	0,065	91,068	82,23809751	99,89790249
**FN_4**	**Yield**	null	Valencia	2001	4	4	3,91	0,105	103,101	97,63486989	108,5671301
**TFW_2**	**Yield**	null	Valencia	2001	2	2	2,71	0,088	87,148	80,62591293	93,67008707
**gFN_2**	Yield	null	Murcia	2007	2	2	2,36	0,055	22,387	10,33969771	34,43430229
**gFN_3**	Yield	null	Murcia	2007	3	3	2,48	0,058	93,904	82,47983403	105,328166
**gFN_5**	Yield	null	Murcia	2007	5	5	2,34	0,054	61,28	49,00959952	73,55040048
**gFN_6**	Yield	null	Murcia	2007	6	6	4,77	0,115	38,954	33,19224673	44,71575327
**gFW_1**	Yield	null	Murcia	2007	1	1	2,58	0,078	66,468	57,97310736	74,96289264

**gTFW_3**	Yield	null	Murcia	2007	3	3	2,78	0,064	95,71	85,35684959	106,0631504
**gTFW_6**	Yield	null	Murcia	2007	6	6	2,65	0,06	38,954	27,91063957	49,99736043
**gTFW_2**	Yield	null	Murcia	2007	2	2	2,28	0,052	21,511	8,768661038	34,25333896
**FL_2.1_C**	**Reproductive**	null	Valencia	2001	2	2	3,56	0,066	1,43	-0,488	10,12611609
**FL_2.2_C**	**Reproductive**	null	Valencia	2001	2	2	3,54	0,066	57,146	48,44988391	65,84211609
**FL_5_C**	**Reproductive**	null	Valencia	2001	5	5	3,23	0,06	38,8	29,2342723	48,3657277
**FL_1.2_C**	**Reproductive**	null	Valencia	2001	1	1	3,14	0,058	101,743	91,84741962	111,6385804
**FL_1.1_C**	**Reproductive**	null	Valencia	2001	1	1	2,25	0,04	47,899	33,55040845	62,24759155
**FR_1_C**	**Reproductive**	null	Valencia	2001	1	1	4,42	0,124	46,285	41,65642208	50,91357792
**FR_5_C**	**Reproductive**	null	Valencia	2001	5	5	2,16	0,06	20,502	10,9362723	30,0677277
**FS_1_C**	**Reproductive**	null	Valencia	2001	1	1	2,38	0,067	49,836	41,26967669	58,40232331
**FS_6_C**	**Reproductive**	null	Valencia	2001	6	6	2,1	0,059	60,181	50,45314132	69,90885868
**L_Cl_2_C**	**Ion**	null	Valencia	2001	2	2	2,9	0,078	77,394	70,03574792	84,75225208
**L_Cl_1_C**	**Ion**	null	Valencia	2001	1	1	2,25	0,06	37,057	27,4912723	46,6227277
**FL_1**	**Reproductive**	null	Valencia	2001	1	1	6,88	0,109	48,533	43,26746182	53,79853818
**FL_8**	**Reproductive**	null	Valencia	2001	8	8	3,86	0,058	77,183	67,28741962	87,07858038
**FL_7**	**Reproductive**	null	Valencia	2001	7	7	3,32	0,049	38,818	27,10486404	50,53113596
**FL_5**	**Reproductive**	null	Valencia	2001	5	5	2,45	0,036	38,8	22,8571205	54,7428795
**FL_2**	**Reproductive**	null	Valencia	2001	2	2	2,11	0,031	51,921	33,40668832	70,43531168
**FR_4**	**Reproductive**	null	Valencia	2001	4	4	5,45	0,148	102,356	98,47800228	106,2339977
**FS_4**	**Reproductive**	null	Valencia	2001	4	4	3,46	0,099	102,356	96,55858927	108,1534107
**L_Na_7_C**	**Ion**	null	Malaga	2003	7	7	6,81	0,1581	39,465	35,83474281	43,09525719
**L_Na_3_C**	**Ion**	null	Malaga	2003	3	3	4,61	0,103	38,739	33,16673144	44,31126856
**L_Na_10_C**	**Ion**	null	Malaga	2003	10	10	2,59	0,051	37,097	25,84320271	48,35079729
**KNa_7_C**	**Ion**	null	Malaga	2003	7	7	5,43	0,158	39,465	35,83244518	43,09755482
**KNa_5_C**	**Ion**	null	Malaga	2003	5	5	2,33	0,061	8,953	0	18,36191249
**L_Na_7**	**Ion**	null	Malaga	2003	7	7	14,16	0,353	39,963	38,33709727	41,58890273
**L_Na_3**	**Ion**	null	Malaga	2003	3	3	2,46	0,046	91,373	78,89596387	103,8500361

**L_K_7**	**Ion**	null	Malaga	2003	7	7	18,22	0,426	39,963	38,61571441	41,31028559
**L_K_8**	**Ion**	null	Malaga	2003	8	8	2,44	0,043	0	0	13,34752702
**L_K_3**	**Ion**	null	Malaga	2003	3	3	2,37	0,042	92,566	78,90067471	106,2313253
**KNa_7**	**Ion**	null	Malaga	2003	7	7	8,13	0,208	48,527	45,76765547	51,28634453
**KNa_8**	**Ion**	null	Malaga	2003	8	8	2,51	0,058	19,121	9,225419621	29,01658038
**TNa_7**	**Ion**	null	Malaga	2003	7	7	11,67	0,24	39,963	37,57156808	42,35443192
**TNa_4**	**Ion**	null	Malaga	2003	4	4	3,75	0,067	49,335	40,76867669	57,90132331
**TNa_3**	**Ion**	null	Malaga	2003	3	3	2,33	0,041	92,561	78,5623741	106,5596259
**NaLS_7**	**Ion**	null	Malaga	2003	7	7	4,15	0,166	39,963	36,50550806	43,42049194
**gRa2WC_7**	**Water**	null	Murcia	2007	7	7	3	0,063	93,633	83,11551387	104,1504861
**gRa2WC_5**	**Water**	null	Murcia	2007	5	5	2,57	0,054	60,651	48,38059952	72,92140048
**gRa2WC_9**	**Water**	null	Murcia	2007	9	9	2,47	0,051	45,68	32,68781125	58,67218875
**gRa2WC_3**	**Water**	null	Murcia	2007	3	3	2,27	0,047	94,404	80,30609306	108,5019069
**gRa2WC_11**	**Water**	null	Murcia	2007	11	11	2,18	0,045	83,705	68,98051942	98,42948058
**gLl2WC_5**	**Water**	null	Murcia	2007	5	5	7,59	0,148	59,492	55,01496199	63,96903801
**gLl2WC_7**	**Water**	null	Murcia	2007	7	7	6,21	0,118	71,936	66,32073198	77,55126802
**gLl2WC_1**	**Water**	null	Murcia	2007	1	1	3,21	0,058	47,126	35,70183403	58,55016597
**gLl2WC_2**	**Water**	null	Murcia	2007	2	2	3,11	0,056	63,517	51,68482811	75,34917189
**gLl2WC_9**	**Water**	null	Murcia	2007	9	9	2,28	0,04	115,112	98,54695935	131,6770407
**gLl2WC_10**	**Water**	null	Murcia	2007	10	10	2,12	0,037	83,176	65,26784795	101,0841521
**gLl2WC_3**	**Water**	null	Murcia	2007	3	3	2,01	0,035	94,404	75,47252497	113,335475
**gRa5WC_3**	**Water**	null	Murcia	2007	3	3	3,95	0,103	94,404	87,9709745	100,8370255
**gRa5WC_7**	**Water**	null	Murcia	2007	7	7	3,27	0,084	104,896	97,0078854	112,7841146
**gLl5WC_7**	**Water**	null	Murcia	2007	7	7	6,33	0,127	71,936	66,71866436	77,15333564
**gLl5WC_5**	**Water**	null	Murcia	2007	5	5	5,23	0,102	59,492	52,99590563	65,98809437
**gLl5WC_3**	**Water**	null	Murcia	2007	3	3	4,47	0,086	94,404	86,69932993	102,1086701
**gLl5WC_9**	**Water**	null	Murcia	2007	9	9	3,32	0,063	115,112	104,5945139	125,6294861
**gLl5WC_2**	**Water**	null	Murcia	2007	2	2	2,99	0,056	69,512	57,67982811	81,34417189

**gLl5WC_1**	**Water**	null	Murcia	2007	1	1	2,51	0,047	49,836	35,73809306	63,93390694
**gLl2_Na_5**	**Ion**	null	Murcia	2007	5	5	2,51	0,0422	61,28	45,57853967	76,98146033
**gLl2_Na_3**	**Ion**	null	Murcia	2007	3	3	4,22	0,026	35,948	10,46332208	61,43267792
**gLl2_Na_7**	**Ion**	null	Murcia	2007	7	7	2,6	0,0247	70,714	43,88802324	97,53997676
**gLl5_Na_10**	**Ion**	null	Murcia	2007	10	10	5,93	0,115	81,863	76,10124673	87,62475327
**gLl5_Na_5**	**Ion**	null	Murcia	2007	5	5	5,73	0,111	61,28	55,31061598	67,24938402
**gLl5_Na_1**	**Ion**	null	Murcia	2007	1	1	4,62	0,088	47,126	39,59643607	54,65556393
**gLl5_Na_7**	**Ion**	null	Murcia	2007	7	7	4,25	0,079	69,714	61,32663765	78,10136235
**gLl5_Na_2**	**Ion**	null	Murcia	2007	2	2	3,03	0,056	63,517	51,68482811	75,34917189
**gLl5_Na_3**	**Ion**	null	Murcia	2007	3	3	2,4	0,043	47,099	31,68965986	62,50834014
**gLl5_Na_9**	**Ion**	null	Murcia	2007	9	9	2,29	0,042	71,915	56,13877081	87,69122919
**gLl5_K_2**	**Ion**	null	Murcia	2007	2	2	2,65	0,087	31,93	24,31388936	39,54611064
**gLl5_K_11**	**Ion**	null	Murcia	2007	11	11	2,45	0,08	92,942	84,65947967	101,2245203
**gLl2_Ca_6**	**Ion**	null	Murcia	2007	6	6	2,86	0,082	84,016	75,93549237	92,09650763
**gLl2_Ca_7**	**Ion**	null	Murcia	2007	7	7	2,69	0,077	99,075	90,46978408	107,6802159
**gLl2_Ca_8**	**Ion**	null	Murcia	2007	8	8	2,50	0,071	102,047	92,71458273	111,3794173
